# Correlation between tear levels of vascular endothelial growth factor and vitamin D at retinopathy of prematurity stages in preterm infants

**DOI:** 10.1038/s41598-023-43338-w

**Published:** 2023-09-27

**Authors:** Ponnalagu Murugeswari, Anand Vinekar, S. Grace Prakalapakorn, Venkata Ramana Anandula, Murali Subramani, Tanuja Arun Vaidya, Archana Padmanabhan Nair, Chaitra Jayadev, Arkasubhra Ghosh, Govindasamy Kumaramanickavel, Rohit Shetty, Debashish Das

**Affiliations:** 1 Stem Cell Research Lab, GROW Lab, Narayana Nethralaya Foundation, Narayana Nethralaya Eye Hospital, 258/A Bommasandra Industrial Area, Bangalore, Karnataka 560099 India; 2 Department of Pediatric Retina, Narayana Nethralaya Eye Institute, Bangalore, India; 3 Department of Ophthalmology, Duke University, Durham, USA; 4 Department of Pediatrics, Duke University, Durham, USA; 5 Department of Molecular Diagnostics and Laboratory Services, Narayana Nethralaya Eye Institute, Bangalore, Karnataka India; 6 GROW Lab, Narayana Nethralaya Foundation, Bangalore, India; 7 Department of Vitreoretinal Services, Narayana Nethralaya Eye Institute, Bangalore, Karnataka India; 8 Department of Cornea and Refractive Surgery, Narayana Nethralaya Eye Institute, Bangalore, Karnataka India

**Keywords:** Translational research, Paediatric research

## Abstract

Deregulation of vascular endothelial growth factor (VEGF) levels leads to retinopathy of prematurity (ROP). Vitamin D (VIT-D) is known to regulate VEGF in an oxygen dependent manner. The purpose of this study was to correlate tear levels of VEGF and VIT-D with different ROP stages in preterm infants. In this prospective cross-sectional study, we enrolled 104 pre-term infants. They were grouped into: Group-1 (Classical ROP) and Group-2 (Aggressive ROP), which were further subdivided into Group-1A (progressing), Group-1B (regressing), Group-2A (pre-treatment), and Group-2B (post-treatment). Tear VEGF and VIT-D levels and their association with different ROP stages were assessed. Stage 1 and stage 2 had higher whereas stage 3 had lower VEGF levels in Group-1B compared to Group-1A. Stage 1 and stage 3 showed higher levels of VIT-D with no difference in stage 2 in Group-1B compared to Group-1A., Group-2B showed higher VEGF and lower VIT-D levels compared to Group-2A. Presence of a positive correlation at an early stage (stage 1) of ROP and a negative correlation at a more advanced stage (stage 3) of ROP with VIT-D and VEGF implies stage-specific distinct signaling crosstalk. These findings suggest that VIT-D supplementation may have the potential to modify the course and outcome of ROP.

## Introduction

Retinopathy of prematurity (ROP) remains the leading cause of bilateral blindness in preterm infants^1,2^. Although multifactorial in aetiology, unmonitored and concentrated oxygen exposure during the early postnatal period is believed to set up a cascade of vascular events in the retina. This cascade leads to vaso-attenuation in the early stage and vascular proliferation in the late stage of ROP development^1,3,4^. Several angiogenic factors have been implicated but vascular endothelial growth factor (VEGF) and angiogenin are of paramount importance^5,6^.

The complexity of ROP development could be driven by low levels of physiologic VEGF in the early stage, and higher levels of pathologic VEGF in the late stage of ROP. Differential oxygen saturation levels in the early and late stages of ROP must also be considered^6–8^. The biological plausibility that restoration of normal levels of physiologic VEGF might prevent the development or progression of ROP is therefore an intuitive assumption. Likewise, the reduction of pathologic levels of VEGF in late stages of ROP may prevent disease progression or worsening. Hence, exploring modalities to replenish ‘deficient’ VEGF levels in the early stage and to dampen, mitigate or annul pathologic VEGF levels in the late stages of ROP might aid in preventing the development and/or progression of ROP.

Pre-term babies are often deficient in micronutrients. Identifying an appropriate nutraceutical with the potential to regulate VEGF levels under various oxygen levels would provide a novel treatment for infants at risk for ROP. Vitamin D (VIT-D) is a nutrient known to modulate inflammation, angiogenesis, oxidative stress, and fibrosis in humans^9–11^. In preterm infants, VIT-D insufficiency has been reported to be associated with respiratory distress syndrome^12^, while an appropriate dosage of VIT-D supplementation has been shown to promote growth and increase bone density^13–15^. VIT-D binding domains are present in the promoter regions of VEGF^16^. Thus, VIT-D supplementation might aid in restoring VEGF levels, especially in pathologic conditions secondary to low VEGF levels.

Tears provide an ideal tool for estimating biomarkers and molecular signatures of ocular vascular changes in health and disease^17^. Our group has shown that at the first visit lower levels of VEGF and corresponding higher levels of angiogenin were detected in tears of infants with ROP compared to infants without ROP^5^. Additionally, the ratios of angiogenin to birth weight or gestational age or VEGF levels could distinguish preterm infants with from those without ROP^5^. We have previously shown a positive correlation between VIT-D in serum and tears of adults^18^. The aim of this study was to investigate tear levels of VEGF and VIT-D and their association with different ROP stages in preterm infants.

## Results

### Baseline characteristics of preterm infants:

**Figure 1:**
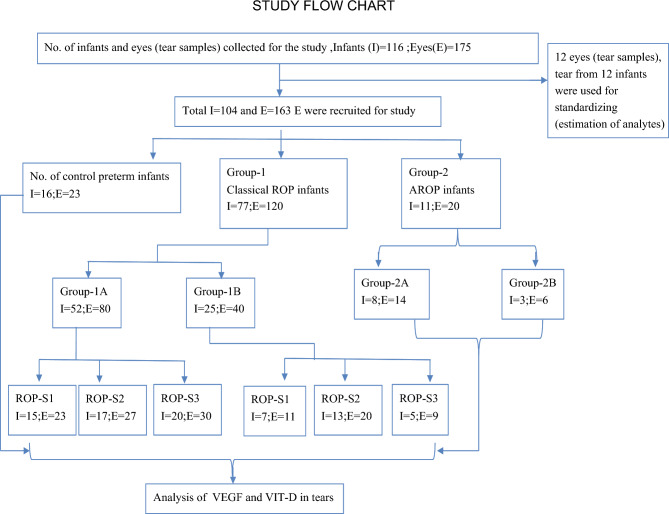
Study Flow chart showing the number of infants (I) and eyes (E)included in this study and grouping of tear samples by clinical stage of ROP on the date of sample collection. AROP, aggressive retinopathy of prematurity; Group-1A, infants/eyes with progression in ROP from its preceding screening visit; Group-1B, infants/eyes with regression in ROP(spontaneous or after laser treatment)from its preceding screening visit; Group-2A, AROP infants/eyes prior to treatment; Group-2B, AROP infants/eyes after laser treatment; ROP, retinopathy of prematurity; S1, stage 1 ROP; S2, stage 2 ROP; S3, stage 3 ROP; VEGF, vascular endothelial growth factor; VIT-D, Vitamin-D.

During the 2-year study period (November 2016–December 2018), 116 preterm infants (175 eyes) fulfilled the inclusion criteria. Twelve infants (12 eyes) were used for standardizing the quantification of tear analytes by ELISA and cytometric bead array. Thus, study analyses were performed on the remaining 104 infants (163 eyes): 77 infants (120 eyes) with classical ROP (Group-1), 11 infants (20 eyes) with AROP (Group-2), and 16 control infants (23 eyes) (Fig. 1). In Group-1B infants, ROP was regressing either spontaneously (n = 17) or after laser therapy (n = 8). Compared to controls, all disease groups (i.e. Group-1A, Group-1B, Group-2A, Group-2B) had statistically significant lower GA and BW. Whereas only Group-1A and Group-2A (but not Group-1B and Group-2B) had statistically significant lower postmenstrual age (PMA) at time of tear sample collection (Supplementary Table 1). The median VEGF and VIT-D were apparently low in controls than progressing Group-1A ROP. In regressing Group-1B ROP VEGF and VIT-D were higher than controls though not significant (Supplementary Table 1).

Following the combined group analysis, the baseline characteristics for each ROP stage in Group-1A and Group-1B infants are detailed in Table 1. Among Group-1A infants, GA and BW were significantly higher in those with stage 1 compared to stage 2 or stage 3 ROP. Comparing infants in Group-1A and Group-2A, PMA was significantly higher in Group-1A infants with stage 1, 2, and 3 ROP whereas GA and BW were significantly higher only among those in Group-1A with stage 1ROP (Table 1). Comparing Group-1B and Group-2B infants, GA was significantly higher in Group-1B infants with ROP stage 1 and 2 (Table 1).

**Table 1:** Baseline clinical characteristics of Group-1 ROP and Group-2 AROP preterm infant patients.

Group-1A (Progressing ROP)	ROP-S1	ROP-S2	ROP-S3	Group-2A (AROP)	*P* values			*P* values		
Characteristics										
					S1 VSS2	S1 VSS3	S2 VS S3	Group-2A VS S1	Group-2A VS S2	Group-2A VS S3
No. of Eyes (No. of infants)	23 (15)	27 (17)	29 (20)	14 (8)						
GA (weeks) (mean ± SE)	31.9 ± 0.377	29.9 ± 0.310	29.6 ± 0.26	28.9 ± 0.178	0.0002	< 0.0001	0.5374	< 0.0001	0.0632	0.1790
PMA (weeks) at time of sample collection (mean ± SE)	38.8 ± 0.91	37.5 ± 0.8	36.7 ± 0.37	34.2 ± 0.40	0.2040	0.1293	0.8205	< 0.0001	0.0015	< 0.0001
BW (g) (mean ± SE)	1707 ± 71.1	1334 ± 71.7	1279 ± 51.3	1354 ± 31.6	0.0024	< 0.0001	0.9188	< 0.0001	0.4155	0.3055
Gender (%)										
Male	60.8	62.9	58.6	50						
Female	39.1	37.0	41.3	50						
GA (weeks) (%)										
≤ 30	26.0	59.2	75.8	92.8						
30.1–33.9	26.0	40.7	24.0	7.1						
34.1–37.9	48.8	0	0	0						
BW (g) (%)										
≤ 750	0	3.7	6.8	0						
750.1– 1000.9	4.3	25.9	6.8	0						
1000.1–1500.9	26.0	40.7	72.4	100						
1500.1–2000	47.8	29.6	13.7	0						

GROUP-1B(Regressing ROP)	ROP-S1	ROP-S-2	ROP-S3	Group-2B (AROP)	S1 VSS2	S1 VSS3	S2 VS S3	Group-2B VS S1	Group-2B VS S2	Group-2B VS S3
No. of Eyes (No. of infants)	11 (7)	20 (13)	9 (5)	6 (3)						
GA (weeks) (mean ± SE)	32 ± 0.19	30.7 ± 0.38	30.6 ± 0.72	29 ± 0.6	0.0707	0.1503	0.7167	0.0005	0.0250	0.0919
PMA (weeks) at time of sample collection, (mean ± SE)	39.1 ± 0.39	41.3 ± 0.63	40.6 ± 0.7	38.0 ± 2.0	0.0615	0.3313	0.5968	0.3832	0.0853	0.2007
BW(g) (mean ± SE)	1450 ± 124	1279 ± 61.3	1398 ± 77.3	1300 ± 63.2	0.1920	0.6455	0.3818	0.5020	0.8238	0.3101
Gender (%)										
Male	81.8	60	66.6	66.6						
Female	18.1	40	33.3	33.3						
GA (weeks)(%)										
≤ 30	0	55	33.3	66.6						
30.1– 33.9	100	45	66.6	33.3						
34.1–37.9	0	0	0	0						
BW (g) (%)										
≤ 750	0	5	0	0						
750.1–1000.9	27.2	10	0	0						
1000.1–1500.9	18.1	70	77.7	100						
1500.1–2000	54.5	15	22.2	0						

### Levels of VEGF and VIT-D in ROP stages

**Figure 2:**
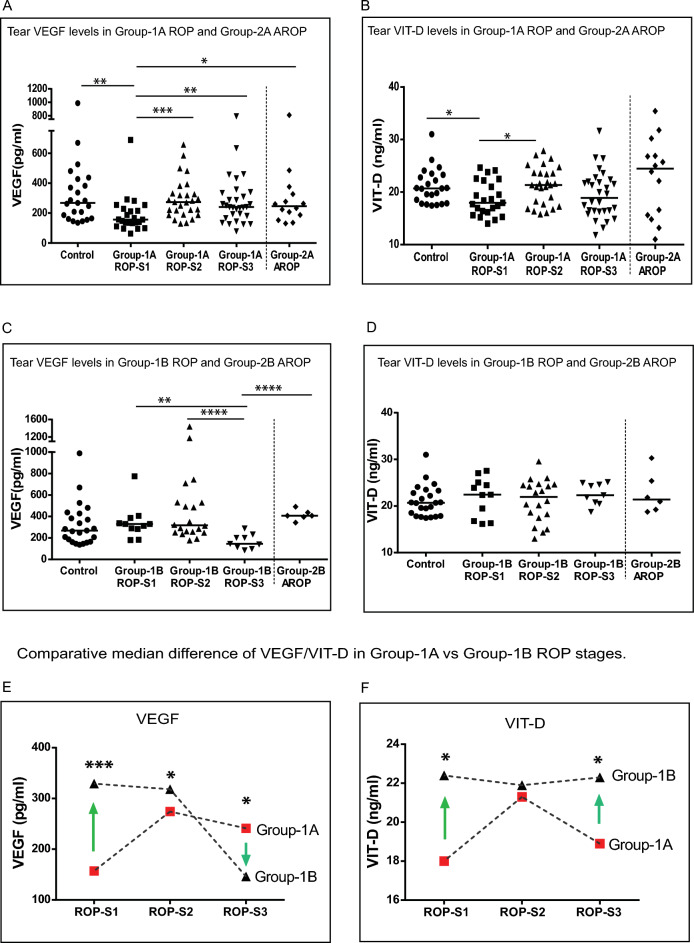
Comparison of tear vascular endothelial growth factor (VEGF) and Vitamin-D (VIT-D) levels in Control, Group-1 (Classical Retinopathy of Prematurity [ROP]) infants by disease activity (i.e. progressing [Group-1A] and regressing [Group-1B]) and ROP stage, and Group-2 (aggressive ROP [AROP]) infants grouped by those prior to treatment (Group-2A) or post-laser treatment (Group-2B). Scatter plots showing the median tear levels of (A and C), VEGF and (B and D) VIT-D. Comparative difference of median tear levels of (E) VEGF and (F) VIT-D between Group-1A and Group-1B infants by ROP stage shows the median difference *p < 0.05, **p < 0.01, ***p < 0.001, ****p < 0.0001.

Followed by the clinical characteristics, the molecular factors VEGF and VIT-D were analyzed in each stages of progressing group-1A and regressing group-1B. We observed that tear VEGF levels in progressing stage1 ROP were significantly lower compared to control, progressing stage 2, stage 3 ROP, and Group-2A AROP (Fig. 2A, Supplementary Table 2). Tear VIT-D levels in progressing stage 1 ROP was significantly lower than its progressing ROP S2 and controls (Fig. 2B, Supplementary Table 2). Similarly, we then compared the tear VEGF and VIT-D levels of control with the stages (stage 1, 2, 3) of regressing Group-1B and Group-2B AROP. Tear VEGF levels in the regressing stage 3 ROP was significantly lower than its regressing stage1 and stage2 ROP and Group-2B APROP (Fig. 2C, Supplementary Table 2). In regressing ROP stages the tear VIT-D levels showed no statistical differences among regressing stages (ROP-S1, ROP-S2, ROP-S3) controls and Group-2B AROP (Fig. 2D). The median (minimum and maximum) values of VEGF and VIT-D and the P values comparing the different Groups, stages and controls are shown in Supplementary Table 2.

We then wanted to find if there was any difference of tear VEGF and VIT-D levels between the stages of progressing Group-1A and stages of regressing Group-1B. Results revealed a 2.09-fold higher ratio (*p* = 0.0001) among those with stage1 ROP, a 1.16-fold higher ratio (*p* = 0.0457) among those with stage 2 ROP, and a 0.6-fold lower ratio (*p* = 0.0183) among those with stage3 ROP (Fig. 2E). Comparison of median tear VIT-D levels revealed a 1.24-fold higher ratio (*p* = 0.0456) among those with stage 1 ROP and a 1.17-fold higher ratio (*p* = 0.0345) among those with stage3 (Fig. 2F). Additionally, we also wanted to determine if there existed any difference of tear VEGF and VIT-D levels among pretreatment Group-2A AROP and post laser treated Group-2B AROP. The median tear VEGF levels revealed a 1.64-fold higher ratio (*p* = 0.0392) (Supplementary Fig. 1A) and of median tear VIT-D levels revealed a 0.87-fold lower ratio (*p* = 0.0117) (Supplementary Fig. 1^1^) in Group-2B compared to Group-2A.

### Correlation of tear levels of VEGF and VIT-D in progressing and regressing stages of ROP and AROP

**Figure 3:**
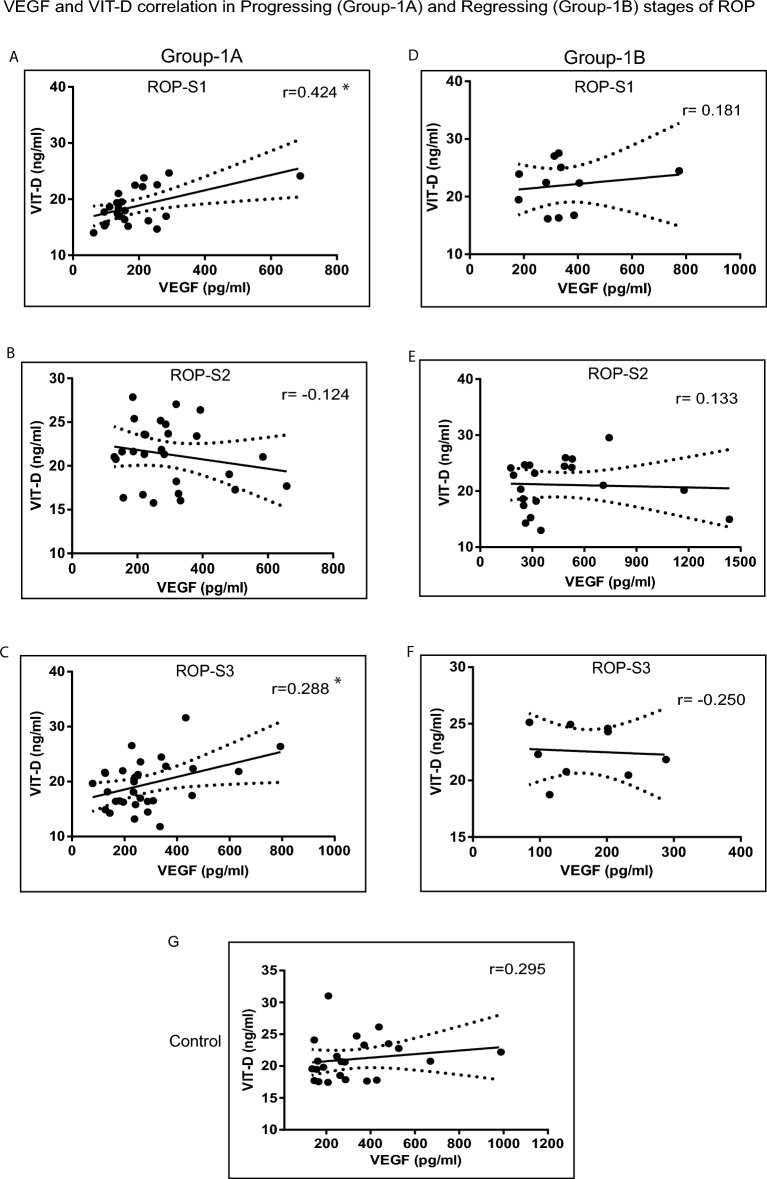
Tear vascular endothelial growth factor (VEGF) and Vitamin-D (VIT-D) levels and their correlation between progressing Group-1A and regressing Group-1B infants by retinopathy of prematurity (ROP) stage. Spearman’s rank correlation for VEGF and VIT-D (A-C) in progressing stage-1, stage-2 and stage-3 ROP; (D-F) in regressing stage-1, stage-2 and stage-3 ROP;and (G) Control. *p < 0.05.

As a next step we wanted to evaluate if there was any correlation of the tear VEGF and VIT-D levels in stages of progressing Group-1A. A significant positive correlation was observed for VEGF and VIT-D in progressing stage 1(*p* = 0.021) and Stage-3 (exact *p* = 0.060) ROP (Figure-3 A and C).Stage-2 progressing ROP showed an apparent negative correlation (*p* = 0.268) (Fig. 3B). In regressing ROP (Group-1B), though not significant ROP-S1 (*p* = 0.297) and ROP-S2 (*p* = 0.286) showed a positive correlation and ROP-S3 showed a negative correlation (*p* = 0.260)(Fig. 3D–F). Controls also showed a positive correlation for VEGF and VIT-D (*p* = 0.085) (Fig. 3G). The Group-2A (*p* = 0.222) and Group-2B (*p* = 0.177) showed a negative correlation (Supplementary Fig. 1C,D).

To determine the relationship of VEGF and VIT-D with GA or BW, we carried out multivariable analysis. In progressing group (Group-1A), the VEGF association with BW and GA showed a statistical significance (*P* = 0.023), though the odds ratio is near 1 (0.995). Similarly, the VIT-D association with BW and GA showed a similar pattern like VEGF with statistical significance (*P* = 0.016) and odds ratio near 1(0.83). In regressing ROP (Group-1B) the BW and GA category though showed an odds ration > 1, no significance was observed. This may be due to smaller sample size and wider confidence interval **(**Supplementary Table 3).

### Discussion

ROP is one of the leading causes of blindness in preterm infants. The present treatment paradigm revolves around anti-VEGF and laser treatment. In an effort to explore potential non-invasive mode of disease management, we investigate a correlation of tear VIT-D and VEGF levels for different stages of ROP. In ocular disease management, tear acts as a non-invasive tool for assaying cytokines and growth factors. To our knowledge this is the first clinical study that reports the association between VEGF and VIT-D in tears of premature infants with ROP. Low levels of serum 25(OH) D have been implicated in low BW preterm infants, respiratory morbidity, and development of ROP^19,20^. The low VIT-D levels in preterm infants may be due to insufficient transfer from maternal source through placenta in the third trimester^21,22^. The lower levels of VIT-D in Group-1A is also dependent on the mothers VIT-D intake^23^. The VEGF physiologic and pathologic role in retinal angiogenesis has been reported^24^. Acting upstream of VEGF regulation, VIT-D and its receptors modulate retinal angiogenesis^16,25^.

We found that among those with progressing classical ROP (i.e. Group-1A), those with stage 1 ROP had lower tear levels of VEGF and VIT-D levels compared to those with higher stage ROP, APROP, and controls. Interestingly, in the regressing classical ROP (i.e. Group-1B) infants the VIT-D and VEGF levels were higher in stage1 ROP whereas in stage3 ROP, VIT-D levels were higher while VEGF levels were lower compared to progressing classical ROP (i.e. Group-1A). The source for the higher levels of VIT-D in the tears of regressing ROP (Group-1B) of stage-1 and stage-3 infants’ may be from maternal breast milk or formula milk^26^. This could also be an outcome driven by increase of VIT-D assimilation following intake. It also needs to be stated that in spite of breastmilk or formula milk there exists VIT-D deficiency in the preterm infants. Studies have emphasized the need for supplementation to maternal breastfeed in order to prevent VIT-D deficiency in infants^27,28^. Supplementation of VIT-D in preterm infants has shown improvement in the linear growth and bone density^15^. In a mice supplementation of VIT-D promoted vascular invasion and endo-chondral ossification by regulating VEGF and matrix metalloproteinase-9^29^.

Further, in this study postnatally infants were feed with breastmilk or formula milk. The VIT-D levels in human milk (40 IU/L; 15–26 IU/L) and formula milk (400 IU/L) may differ^23,30–32^. For a healthy infant 50 nm/L of standard 25 (OH) D concentration is required. Studies, state that breast fed infants VIT-D may be inadequate to reach the normal value. Moreover, formula fed infants need to take 1 L of milk every day, for an intake of 400 IU/L VIT-D. In newborn infants it is not possible to consume 1 L of milk/day, which also leads to inadequate amount of VIT-D^32^. Previous study from our group, stated that tear VIT-D levels are higher than serum Vit-D levels, suggesting it may have its own relevance for certain ocular pathogenesis and disease severity^18^. In this study, tear VIT-D levels might be an outcome of absorption and intake of each infant. This study did not compare with any standard cutoff values. Though breastfed/formula milk VIT- D may be not adequate for all infants, however, it could be one of the main sources of VIT-D in this study. In the regressing Classical ROP (i.e. Group-1B) infants, the increased VEGF in tears of stage1 ROP infants might have resulted from either maternal breast milk or due to the physiological increase with increasing gestational age^33^, while in the stage3 ROP infants, the lower levels of VEGF can be attributed to laser treatment^34^.

In our previous study, we demonstrated that hyperoxia-conditioned primary retinal pigment epithelial (RPE) cells secreted low levels of VEGF^35^. Further we functionally demonstrated an incomplete tube formation in vitro, which supports our current findings of lower tear VEGF levels in stage1 of classical progressing ROP. In our in vitro study, VIT-D supplementation increased VEGF levels in the primary RPE cells and improved endothelial tube formation^36^. At a molecular level it has been shown that VIT-D has a binding affinity in the promoter region of VEGF and induces the regulation of VEGF^16^. Further studies investigating the role of VIT-D supplementation on stage-1 ROP disease would possibly provide a newer avenue of ROP disease management. It needs to be explored if laser treatment could have influenced the increase in VIT-D levels. Further, the spearman’s rank correlation analysis for VEGF and VIT-D in Stage-3 ROP belonging to Group-1A, showed an inverse correlation. It is also reported that VIT-D acts as an anti-angiogenic factor in hypoxic conditions such as in cancer by regulating the endothelial cells^35,37,38^.

The in vitro hyperoxic condition may be ‘closer’ to stage1 ROP and the hypoxic condition akin to stage3 ROP. It is also reported that VIT-D acts as an anti-angiogenic factor in hypoxic conditions like cancer by regulating the endothelial cells^35,37,38^. Therefore, VIT-D regulating VEGF may be influenced by oxygen saturation levels, as it is acting as a pro-angiogenic factor in hyperoxic conditions and anti-angiogenic in hypoxic conditions.

In this study, stage2 ROP showed variable results and demanding further research. Comparable levels of VEGF in Group-1B ROP stage3 infants further corroborates the possibility that VEGF may play a dual physiological and pathological role depending on the prevailing oxygen saturation condition. Tear analysis of Group-2A infants showed low VEGF and high VIT-D levels compared to Group-2B. This implies that the Group-2B infants need aggressive treatment to reduce the proangiogenic factors. In the present study, we included AROP eyes as a positive control for the VEGF levels. However, further investigations on understanding detailed crosstalk of VIT-D and proangiogenic factors are needed.

Low GA and BW remain the most promising determinants of ROP, that is convincingly restored in patients with resolved ROP^39^. Additionally, studies have revealed that premature infants with high GA and BW have a low risk of developing ROP^40^. In our study cohort we also observed that GA and BW showed high mean levels in the regressing (Group-1B) group in all stages of ROP. Though, VIT-D levels might play a crucial role in occurrence and progression of the disease and its resolution, the pertinent role of BW and GA cannot be undermined. Furthermore, studies can be designed to elaborate the implication of VIT-D with GA/BW to understand the underlying mechanisms.

It is well established that along with ROP, pre term infants can have additional non-ocular complications such as respiratory distress syndrome (RDS), necrotizing enterocolitis (NEC) and intraventricular hemorrhages (IVH). In our study, IVH was detected in just one infant. NEC was observed in eight cases, evenly divided between Group-1A and Group-1B, with four instances in each. Respiratory Distress Syndrome (RDS) was notably prevalent in our study, particularly in Group-1A, where a higher prevalence of RDS corresponded to more severe disease groups. Within progressing Group-1A, 15 out of 23 cases (65.2%) in the control group and 15 out of 23 cases (65.2%) in ROP Stage-1 exhibited RDS, with average tear Vitamin D concentrations of 22.5 ng/mL and 19.9 ng/mL, respectively. ROP Stage-2 showed RDS in 20 out of 27 cases (74.0%), while Stage-3 had RDS in 23 out of 30 cases (76.6%), with average tear Vitamin D concentrations of 21.4 ng/mL and 19.6 ng/mL, respectively.

There are no studies implying tear Vitamin D levels with RDS. All the studies have correlated the serum Vitamin D levels and RDS. There being a positive correlation between the tear and serum Vitamin D levels^18^, it could be assumed that although the control and Stage-1 groups had similar percentages of RDS cases, controls had a higher concentration of Vitamin D compared to ROP Stage-1, which might have acted as a preventive factor in controls. The lower Vitamin D concentration in Stage-1 could potentially contribute to disease progression. However, in Stage-3, where RDS was present in a higher percentage, Vitamin D concentration was lower compared to Stage-2 and equal to Stage-1. The systemic arterial oxygen saturation index might also play a role in regulating Vitamin D levels for development of RDS.

In regressing Group-1B, RDS was prevalent in higher numbers, nearly identical across all groups when compared to controls. Specifically, Group-1B ROP Stage-1 had RDS in 10 out of 11 cases (90.9%), Stage-2 had RDS in 15 out of 20 cases (75%), and Stage-3 had RDS in 7 out of 9 cases (77.7%), with respective Vitamin D concentrations of 21.4 ng/mL, 20.45 ng/mL, and 22.27 ng/mL. A discernible pattern of Vitamin D levels corresponding to RDS was observed in Group-1B. In Group-2A, AROP had RDS in 10 out of 14 cases (71.4%), while Group-2B had RDS in 4 out of 6 cases (66.6%).

A meta-analysis study demonstrates an association between vitamin D levels within 24 h of birth and the risk of bronchopulmonary dysplasia^41^. In a recent study vitamin-D deficiency below 30 ng/mL was associated with increased risk for respiratory distress syndrome^42^. A clinical study demonstrated that vitamin D supplementation significantly reduces serum inflammation factors and the incidence of bronchopulmonary dysplasia in preterm infants^43^. In 2018, Yang et al.^44^ validated that vitamin D deficiency was associated with a higher risk of necrotizing enterocolitis in premature infants. In animal models, Vitamin D has been shown to promote proliferation, inhibit apoptosis, reduce inflammation, and protect the intestinal injury of necrotizing enterocolitis^45,46^. Further, an increased risk of intraventricular hemorrhages is associated with low vitamin-D levels of preterm infants with recommendations for supplementation^47^.

A limitation of this study is lack of longitudinal follow-up, which would have provided patient specific information on the interaction of VEGF and VIT-D over time. Oxygen saturation is a crucial component during retinal vasculature development. Due to ethical and technical limitations, we have not investigated the oxygen saturation in affected eyes. Studies in animal models would provide insight into the role of oxygen in the interaction between VEGF and VIT-D. These findings could alter the treatment course of ROP by providing a better understanding of the interplay between VEGF, VIT-D, and retinal status. For example, VIT-D supplementation during stage 1 ROP might halt ROP progression by regulating VEGF and promoting physiological vascular growth towards the *ora serrata*. Similarly, VIT-D supplementation in laser-treated stage3 ROP eyes may further help regression of severe ROP (i.e. pathologic vascular growth) and promote physiologic vascular growth. Possible routes of VIT-D administration for better bioavailability need to be investigated. We hope that our findings serve as a foundation to explore possible therapeutic potentials of VIT-D supplementation in premature infants at risk for ROP.

## Methods

### Study design

This cross-sectional study was carried out in the Department of Pediatric Retina, Narayana Nethralaya Eye Institute, Bangalore, India between November 2016 and December 2018. This study used the International Classification of ROP (ICROP) revisited and Early Treatment for ROP (ETROP) guidelines for screening, staging and treatment of infants^48–50^. All preterm infants screened for this study followed the methodology of the Karnataka Internet assisted Diagnosis for ROP (KIDROP) program^51,52^. Tear samples were collected from infants following the guidelines of Narayana Nethralaya Ethics Committee and abiding by the tenets of the Declaration of Helsinki. This study was performed with the approval of Narayana Nethralaya Institutional Review Board. Written informed consent was obtained from the parents of all infants included in the study.

### Study population

Preterm infants with a birth weight (BW) of ≤ 2000 g and gestational age (GA) of ≤ 34 weeks were recruited for the study according to the prevailing Indian National ROP screening guidelines^53^ and the inclusion criteria of the KIDROP program^51,52,54,55^. We collected demographic details including BW, GA, gender and postnatal factors. As a part of the KIDROP screening program, wide-field imaging was performed on all preterm infants using the RetCam Shuttle (Natus, CA, USA), or the 3Nethra Neo Camera (Forus Health, India). In this study, we collected tears at one ROP screening visit from preterm infants who presented to the headquarters during any visit of ROP screening and whose parents consented for the study.

### ROP group classification for this study

Based on the clinical stage of ROP on the date of sample collection, tear samples were grouped into two disease groups (Group-1 [Classical ROP], Group-2 [Aggressive ROP (AROP)]) and a control group. Samples in Group-1 were subdivided into ‘progressing’ (Group-1A) or ‘regressing’ disease (Group-1B) depending on the clinical findings prior to and subsequently after the date of tear collection which was ascertained from a retrospective chart review. ‘Progression’ (Group-1A) was defined by an increase or worsening of the disease severity with respect to stage or plus component of the disease compared to the previous screening visit. Infants in progression group were treatment naive up to the date of tear sample collection. ‘Regression’ (Group-1B) was defined as decrease in disease severity or improvement in disease severity with respect to stage or plus component of the disease compared to the previous screening visit which occurred either spontaneously or after laser therapy. Samples in Group-2 were subdivided into pretreatment eyes with AROP (Group-2A) and post laser-treated eyes (Group-2BThe control group had preterm infants who did not develop any ROP throughout their screening visits and whose retinal vessels had reached the *ora serrata*.

For this study, preterm infants were excluded if they had any of the following: stage 4 or 5 ROP, anti-vascular endothelial growth factor (anti-VEGF) treatment, BW > 2000 g, screened after 4 weeks of birth, or who did not fulfill the criteria of the national screening guidelines.

### Tear sample collection and processing

Tear samples were collected from preterm infants at one ROP screening visit using Schirmer strips (Contacare Ophthalmics and Diagnostics, Gujarat, India) before any topical medication. The strips were placed on the conjunctival fornices and removed on wetting of 15 mm or more. The removed strips were then stored in sterile eppendorf tubes at − 80 °C freezer. For the estimation of analytes, the Schirmer’s strips were cut, agitated and incubated for 2 h in phosphate buffered saline (PBS) in sterile Eppendorf tubes at 4 °C. The samples were centrifuged at 8.6 g for 10 min and the supernatant was analyzed for VEGF and VIT-D levels^5^.

### Vitamin-D measurement

Tear VIT-D (25-hydroxyvitamin-D2-D3[25OH-D2 and 25OH-D3]) levels were estimated using a competitive Enzyme-Linked Immuno-sorbent Assay (ELISA; DIA Source, BY, Germany). The tear samples were diluted 1:10 times for the absorbance value to be within the standard range. The standards and samples were analyzed per the manufacturer’s instructions. The substrate reaction product was read with a microplate reader (Bio-Rad, CA, USA) at a wavelength of 540 nm with the reference filter at 670 nm.

### VEGF measurement

Tear VEGF-A levels were quantified using a cytometric bead array (BD biosciences, NJ, USA). The assay was carried out per the manufacturer’s instructions. A 1:5 dilution factor was used for each tear sample. A single bead analyte bead position was acquired, and the beads were read out in a FACS Caliber machine (BD, NJ, USA), using the FCAP array™ software (version 3.0).

### Statistical analysis

GraphPad Prism 6 software was used for all statistical analyses (GraphPad Software, Inc., La Jolla, CA). Mann–Whitney U-test was used to determine the significant difference of VEGF or VIT-D levels by ROP stage. Spearman’s Rank correlation test was conducted to evaluate associations between tear VEGF and VIT-D levels in each stage. The results were considered statistically significant a *P* ≤ 0.05.

### Supplementary Information

Supplementary Information.

## Data Availability

The data supporting the study findings are available on request from the corresponding author.
