# Supplementary material for: Correlation between tear levels of vascular endothelial growth factor and vitamin D at retinopathy of prematurity stages in preterm infants
Source: Sci Rep. 2023 Sep 27;13:16175. doi: 10.1038/s41598-023-43338-w (PMC10533881; doi:10.1038/s41598-023-43338-w)
Supplement: Supplementary file 1 — Supplementary Information. [file 41598_2023_43338_MOESM1_ESM.pdf]

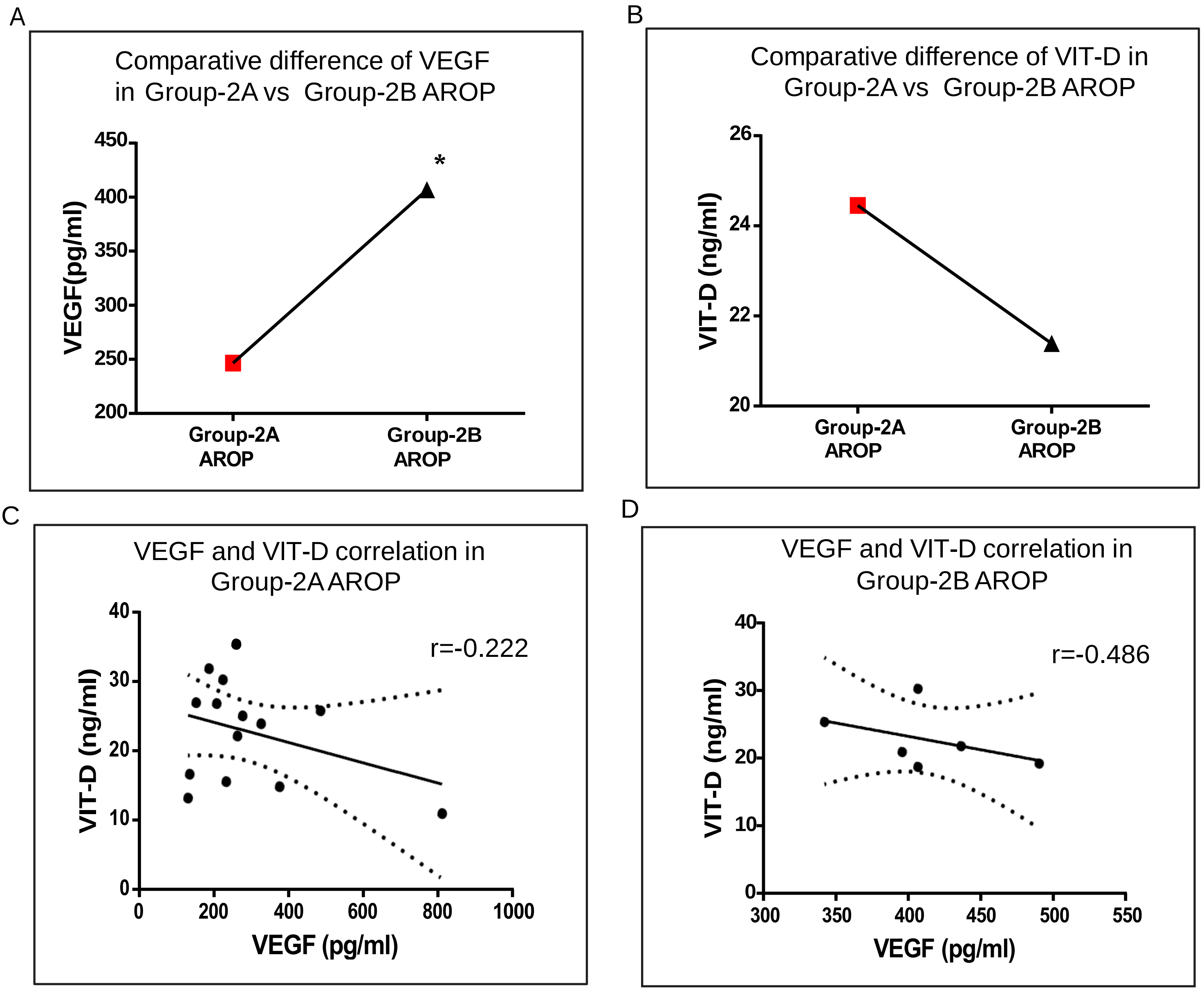

Supplementary Figure-1

Supplementary Table 1: Baseline Clinical Characteristics of Preterm Infants in Group-1, Group-2, and Control Group

CHARACTERISTICS	Control	Group-1A (Pro-gressing ROP)	Group-2A (Pre-treatment AROP)	Group-1B (Re-gressing ROP)	Group-2B (Post-treatment AROP)	P-Value					
						Control vs Group-1A	Control vs Group-2A	Control vs Group-1B	Control vs Group-2B	Group-1A ROP vs Group-2A AROP	Group-1B ROP vs Group-2B AROP
No. of Infants	**16**	**52**	**8**	25	3						
No. of eyes (i.e. tear samples collected)	**23**	**79**	**14**	40	6						
Gestational Age (weeks) (mean ± SE)	**32.5± 0.44**	**30.4± 0.2**	**28.9± 0.178**	**31.03± 0.26**	**29± 0.63**	**<0.0001**	**<0.0001**	**0.0029**	**0.0008**	**0.0056**	**0.0051**
PMA (weeks) at time of tear sample collection (mean ± SE)	**40.5± 0.57**	**37.6± 0.4**	**34.2± 0.40**	**40.5± 0.39**	**38± 2.0**	**<0.0001**	**<0.0001**	**0.8839**	**0.2452**	**<0.0001**	**0.1370**
Birth weight (g) (mean ± SE)	**1719±7 2.7**	**1422± 42.2**	**1354± 31.6**	**1353± 49.1**	**1300± 63.2**	**0.0018**	**0.0003**	**0.0002**	**0.0039**	**0.5669**	**0.7074**
Gender (%)<br>Male<br>Female	 **56.5**<br>**43.4**	 **59.1**<br>**40.8**	 **50**<br>**50**	 **67.5**<br>**32.5**	 **100**<br>**0**						
Vascular endothelial growth factor (pg/ml) Median (min, max)	**268 (136, 988)**	**231 (63.1, 794)**	**247 (131, 812)**	**288.5 (84.3, 1436)**	**406.8 (342.5, 490.2)**	**0.1257**	**0.5579**	**0.6300**	**0.8621**	**0.5375**	**0.039**
Vitamin D (ng /ml) median; (min, max)	**20.7 (17.4, 31.0)**	**19.6 (11.8, 31.6)**	**24.5 (11, 35.4)**	**22.4 (12.9, 29.5)**	**21.3 (18.7, 30.2)**	**0.1788**	**0.4097**	**0.3115**	**0.3840**	**0.1333**	**0.7868**

AROP, aggressive retinopathy of prematurity; Group-1A, infants/eyes with progression in ROP from its preceding screening visit; Group-1B, infants/eyes with regression in ROP (spontaneous or after laser treatment) from its preceding screening visit; Group-2A, AROP infants/eyes prior to treatment; Group-2B, AROP infants/eyes after laser treatment; PMA, postmenstrual age; ROP, retinopathy of prematurity.

Supplementary Table 2: Median tear levels of VEGF and VIT-D in Group-1 (Classical ROP), Group-2 (AROP), and Control Infants

EGF/ VIT-D	Control	ROP-SI	ROP-SII	ROP-SIII	AROP	Control vs ROP-S1	Control vs ROP-S2	Control vs ROP-S3	Control vs AROP	**ROP-S1**vs ROP-S2	**ROP-S1** vs ROP-S3	**ROP-S1**vs AROP
**Group-1A (progressing ROP)**					**Group-2A (AROP)**	P value				P value		
VEGF-Median (Min,Max)	268.3 (135.9, 988.1)	157 (63.1, 689)	274 (130, 658)	241 (78.9, 794)	247 (131, 812)	0.0014	0.9645	0.3848	0.5579	0.0004	0.0065	0.0182
VIT-D Median (Min,Max)	20.7 (17.4, 31)	18 (14,24.7)	21.3 (15.3,27.9)	18.9 (11.8, 31.6)	24.5 (11, 35.4)	0.018	0.6467	0.1180	0.4097	0.0215	0.6922	0.1091
VEGF/ VIT-D	Control	ROP-SI	ROP-SII	ROP-SIII	AROP	Control vs ROP-S1	Control vs ROP-S2	Control vs ROP-S3	Control vs AROP	**ROP-S3**vs ROP-S1	**ROP-S3**vs ROP-S2	**ROP-S3** vs AROP
**Group-1B (regressing ROP)**					**Group-2B (AROP)**	P value				P value		
VEGF-Median (Min,Max)	268.3 (135.9, 988.1)	329.2 (180.9, 774.6)	317.6 (176.2, 1436)	145.7 (84.3, 288.3)	406.8 (342.5, 490.2)	0.3817	0.0012	0.0064	0.0621	0.0012	<0.0001	0.0004
VIT-D Median (Min,Max)	20.7 (17.4, 31)	22.4 (16.2, 27.5)	21.9 (12.9, 29.6)	22.3 (18.7, 25.1)	21.4 (18.7, 30.3)	0.5597	0.7135	0.1028	0.3840	0.9256	0.0.4155	0.8212

AROP-aggressive retinopathy of prematurity; Group-1A, infants/eyes with progression in ROP from its preceding screening visit; Group-1B, infants/eyes with regression in ROP (spontaneous or after laser treatment) from its preceding screening visit; Group-2A, AROP infants/eyes prior to treatment; Group-2B, AROP infants/eyes after laser treatment; ROP-retinopathy of prematurity; AROP-aggressive retinopathy of prematurity; S1, stage 1 ROP; S2, stage 2 ROP; S3, stage 3 ROP; VEGF, vascular endothelial growth factor; VIT-D, Vitamin-D.

Supplementary Table-3

Multivariate analysis of classical ROP groups (Group-1A and Group-1B) for VEGF and VIT-D with gestational age and birth weight.

	Group-1A (Progressing ROP)			Group-1B (Regressing ROP)		
	**OR**	**95% C.I**	**P-Value**	**OR**	**95% C.I**	**P-Value**
**VEGF**	0.995	0.992 – 0.999	**0.023**	1.001	0.999 – 1.004	0.203
Birth Weight (g)						
>2000	--	--	--	--	--	--
1500-2000	1.25	0.09 – 16.18	0.866	0.19	0.19 – 1.96	0.165
1000-1500	2.20	0.14 – 34.45	0.573	1.26	0.11 – 14.89	0.855
750 -1000	8.20	0.28 – 239.09	0.221	--	--	--
≤750	--	--	--	--	--	--
GA (Weeks)						
34.1	--	--	--	--	--	--
30.1-34.0	0.38	0.09 – 1.45	0.157	2.27	0.49 – 10.33	0.290
≤30	--	--	--	--	--	--
**VIT-D**	0.83	0.72 – 0.97	**0.016**	0.97	0.81 – 1.16	0.739
Birth Weight (g)						
>2000						
1500-2000	1.06	0.08 – 14.58	0.967	0.18	0.02 – 2.01	0.163
1000-1500	1.88	0.11 – 31.56	0.660	0.82	0.07 – 9.278	0.870
750 -1000	4.36	0.15 – 125.66	0.391	--	--	--
≤750	--	--	--	--	--	--
GA (Weeks)						
34.1	--	--	--	--	--	--
30.1-34.0	0.40	0.11 – 1.49	0.174	1.56	0.40 – 6.00	0.520
≤30	--	--	--	--	--	--

Representative RetCam images of study groups

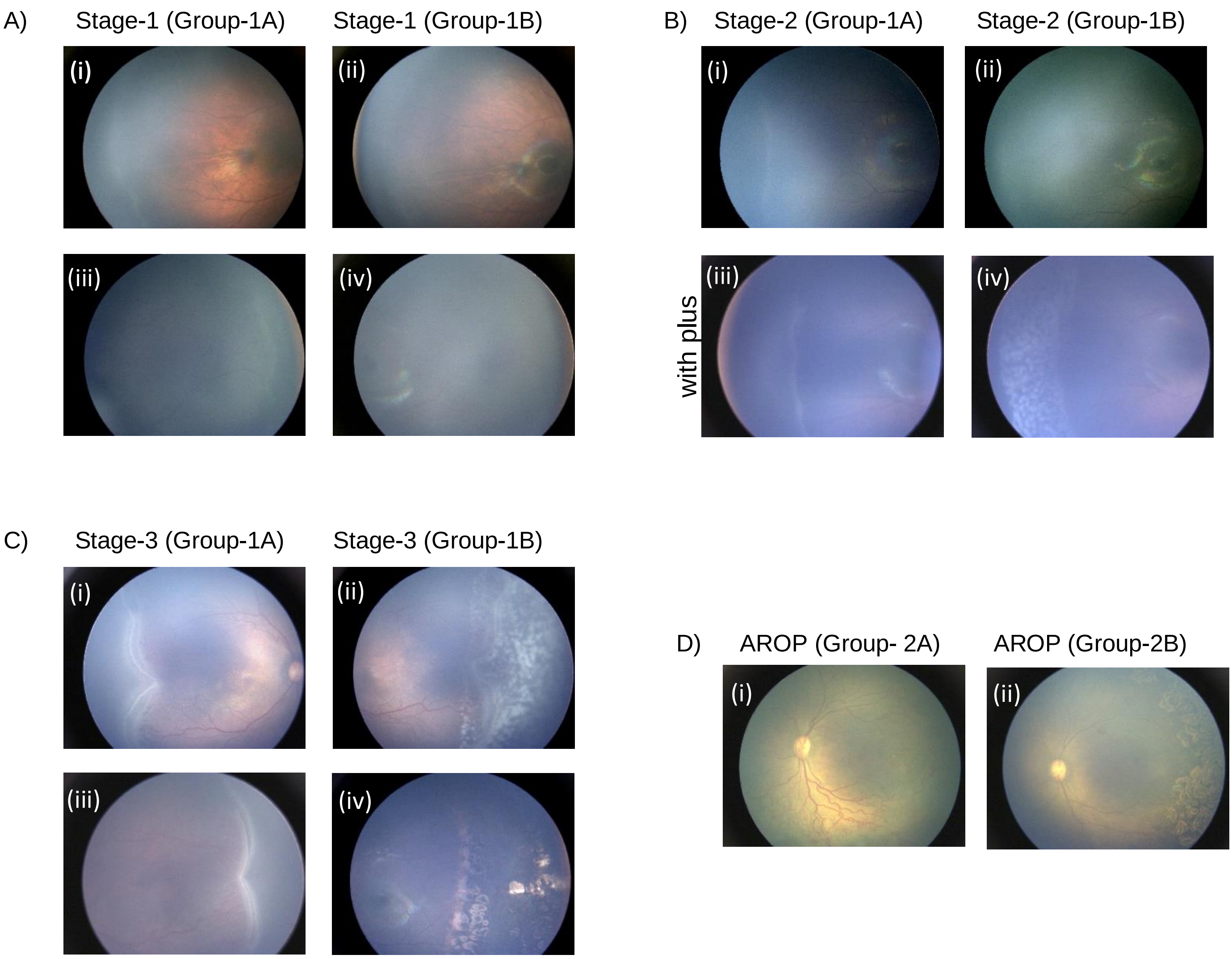

Supplementary Figure 2

Supplementary Figure Legends:

Supplementary Figure- 1

A comparative median difference and correlation of tear levels of VEGF and VIT-D in APROP infants. Graph shows the comparative median difference of high VEGF (A), and low VIT-D (B) in Group-2A compared to Group-2B infants. Spearman's rank correlation-coefficient of VEGF and VIT-D in Group-2A (C), and Group-2B (D) infant's tears.*p<0.05.VEGF-vascular endothelial growth factor; VIT-D-Vitamin-D, ROP-retinopathy of prematurity, AROP-aggressive posterior retinopathy of prematurity.

Supplementary Figure- 2

RETcam Image showing ROP Stage-1 (Fig A) progressing disease (i) and (iii), Stage 1 regressed to mature retina (ii) and (iv). ROP Stage-2 (Fig B) progressing disease (i), regressed spontaneously to normal (ii). Stage-2 with plus progressing disease (iii), regressed after laser treatment (iv). Stage 3 (Fig C) progressing disease (i) and (iii) regressing (after laser treatment) (ii) and (iv). Image showing progressing AROP (Fig D (i)) and regressing after laser treatment (Fig D (ii)).

Supplementary Table Legends:

Supplementary Table-1

Baseline characteristics of preterm infants comparing controls with Group-1A, Group-1B, Group-2A, Group-2B.

Group-1A, infants/eyes with progression in disease condition from its preceding screening visit; Group-1B, infants/eyes with regression in disease condition (spontaneous or after laser treatment)from its preceding screening visit; Group-2A, AROP infants/eyes prior to treatment; Group-2B, APROP infants/eyes with features of residual disease activity from preceding screening visit after laser treatment; ROP-retinopathy of prematurity; AROP-aggressive posterior retinopathy of prematurity; GA-gestational age , BW- birth weight and PMA- post menstrual age. -vascular endothelial growth factor; VIT-D-Vitamin-D,

Supplementary Table-2

Median levels of VEGF and VIT-D in tears of Group-1 (Classical ROP), Group-2 (AROP), and Control Infants

ROP-S1= stage1 ROP, ROP-S2=stage2 ROP, ROP-S3=stage3 ROP.VEGF-vascular endothelial growth factor; VIT-D-Vitamin-D, ROP-retinopathy of prematurity, AROP-aggressive posterior retinopathy of prematurity.Group-1A, infants/eyes with progression in disease condition from its

preceding screening visit; Group-1B, infants/eyes with regression in disease condition (spontaneous or after laser treatment)from its preceding screening visit;Group-2A, AROP infants/eyes prior to treatment; Group-2B, AROP infants/eyes with features of residual disease activity from preceding screening visit after laser treatment;

Supplementary Table-3

Multivariate analysis of classical ROP groups (Group-1A and Group-1B) for VEGF and VIT-D with gestational age and birth weight. ROP- retinopathy of prematurity; AROP- aggressive ROP; VEGF- vascular endothelial growth factor; VIT-vitamin-D
